# Toward relational biosecurity: understanding AI-enabled biology as a connected system

**DOI:** 10.3389/fmicb.2026.1856819

**Published:** 2026-05-14

**Authors:** Michelle Holko

**Affiliations:** 1 International Computer Science Institute, Berkeley, CA, United States; 2 Defensive Biotech, Bethesda, MD, United States; 3 Computercraft, Washington, DC, United States

**Keywords:** AI safety, AI-enabled biology, biosecurity, compositional risk, cyberbiosecurity, human-in-the-loop, socio-technical systems, systems governance

## Abstract

Advances in artificial intelligence (AI) are rapidly expanding what can be predicted and produced in biotechnology, transforming the life sciences into a computational, distributed, and increasingly design-oriented enterprise. At the same time, biosecurity frameworks remain largely organized around the control of known agents, materials, and individual technologies, creating a growing mismatch between emerging capability and current approaches to risk. In AI-enabled biology, capability is increasingly compositional, arising from interactions among data, models, infrastructure, and workflows. Risk no longer resides solely within individual components, but in how they are connected. Safeguards that are effective in isolation may fail when systems are integrated; for example, nucleic acid sequence screening may not capture risks arising from generative systems that explore novel biological space beyond reference frameworks. This Perspective argues for a relational approach to biosecurity that treats interactions between components as explicit objects of design and governance, rather than implicit byproducts of integration. It emphasizes system-level sensing, preservation of context and uncertainty, buffering of perturbations, and alignment with shared values across distributed actors. Rather than replacing existing safeguards, this approach extends them by addressing how they interact within workflows. It introduces the need to represent, measure, and propagate system-level objectives across components, enabling more coherent oversight of compositional workflows. While grounded in biological applications, these challenges extend to AI safety and governance more broadly, where risks emerge from interactions among systems rather than individual models.

## Introduction

Advances in artificial intelligence (AI) are rapidly expanding the space of what can be predicted and produced in biotechnology. Machine learning systems now enable high-accuracy protein structure prediction (Jumper et al., 2021), generative design of novel biomolecules (Madani et al., 2023), and large-scale inference of biological function from sequence data (Rives et al., 2021), transforming the life sciences into a computational, distributed, and increasingly design-oriented enterprise (Häse et al., 2019). At the same time, existing biosecurity frameworks remain largely organized around the control of known agents, materials, and individual technologies (National Academies of Sciences, Engineering, and Medicine, 2018). This creates a widening mismatch between emerging capability and current approaches to risk. As AI-enabled biological systems become more interconnected, capability is increasingly compositional, arising from interactions among models, data, and workflows across scales. In this context, risk no longer resides solely within individual components, but in how they are combined and deployed.

While existing safeguards operate effectively at the level of individual components, they often do not account for how capabilities compose across systems. This can be thought of as compositional risk: risk that arises not from individual components, but from interactions within connected systems. As a result, a system may remain compliant at each step while producing outcomes that would be considered unsafe if evaluated holistically.

This reflects a broader transformation in the life sciences. AI is restructuring biotechnology as a distributed socio-technical system in which distinctions between digital and physical domains are increasingly blurred (Peccoud et al., 2018; Carter et al, 2023). While prior work has examined dual-use risks, access to biotechnological capability, and governance of individual technologies, this Perspective draws on inspiration from the fields of systems biology, cybersecurity, and AI safety to advance three distinct contributions. First, it reframes biosecurity in AI-enabled biology as a problem of governing compositional workflows rather than discrete components. Second, it emphasizes that relationships between components must be explicitly designed, monitored, and governed, rather than treated as secondary effects. Third, it introduces the need for system-level alignment through the representation and propagation of shared values across distributed actors and infrastructures.

This challenge parallels developments in cybersecurity and cyberbiosecurity, where security increasingly depends on visibility, coordination, and information sharing across organizational and technical boundaries. Organizations such as the Bioeconomy Information Sharing and Analysis Center (Bio-ISAC) reflect this shift by emphasizing cyberbiosecurity adoption across AI, biotechnology, biomanufacturing, and data integrity. A relational biosecurity approach extends this logic to AI-enabled biological design by focusing on how safeguards, actors, and workflows interact across the system.

Recent work in AI safety and governance has emphasized that the behavior of AI systems cannot be fully understood by evaluating models in isolation, but must be studied in the context of how they are deployed and interact within broader socio-technical systems (Rahwan et al., 2019; Floridi et al., 2018). There is growing recognition that capabilities, and associated risks, can emerge from the composition of multiple systems, including tool-augmented models and multi-agent workflows. However, existing safety and governance approaches remain largely oriented toward individual models or components. AI-enabled biotechnology provides a concrete and consequential domain in which to examine this challenge, as system outputs are instantiated in physical processes and subject to real-world constraints. These dynamics may manifest differently across deliberate misuse, accidental harm, and structural risk, but in each case the relevant concern is not only the properties of individual components, but how capabilities, incentives, and safeguards interact across workflows.

## AI-enabled biology as a connected system

AI-enabled biotechnology can be understood as a connected socio-technical system composed of interacting layers, including biological data, machine learning models, computational and laboratory infrastructure, and institutional actors. Importantly, this system is not only technical but organizational and institutional. Decisions made in one part of the system may have downstream effects that are not visible to other actors, creating gaps in coordination and accountability. These elements interact dynamically across integration points, with outputs from one layer shaping behavior in others.

This perspective is related to systems biology, which demonstrates that function emerges from interactions across networks, rather than isolated components (Kitano, 2004; Barabási et al., 2011). However, while systems biology provides descriptive tools for analyzing biological systems, it does not directly address how to design or govern engineered socio-technical systems in which responsibility, visibility, and control are distributed across actors. These actors span academia, industry, infrastructure providers, and public institutions, each with distinct incentives, constraints, and visibility into the system. A relational biosecurity approach extends beyond analysis to propose design and governance principles for such systems. As AI becomes embedded in biotechnology workflows, similar dynamics emerge where capability arises from integration rather than from individual tools. At the same time, these interactions introduce new forms of risk. Bias, error, or manipulation in one component can propagate across the system, producing effects that are not visible at the level of any individual model (Peccoud et al., 2018; Rahwan et al., 2019). Understanding these systems therefore requires attention to relationships in addition to individual components.

As a result, the relationships that define system behavior extend beyond technical interfaces to include organizational and institutional interactions. Decisions made in one part of the system, such as in model development, data curation, or infrastructure design, can have downstream implications that are not visible to other actors. For example, biotechnology companies developing models may perform reasonable risk management, but when models from different companies are composed together, novel risks may emerge that are not apparent at the level of any individual organization. This fragmentation reflects historical patterns of specialization, but in the context of increasingly connected systems, it introduces challenges for coordination, accountability, and shared understanding of risk.

## From siloed understanding to system-level insight

While biological systems are increasingly understood as interconnected networks, biotechnology and AI-enabled biotechnology often remain structured around modular representations and task-specific models. These approaches have enabled significant progress, but they can obscure the cross-system interactions that govern system-level behavior.

Research in network physiology and organ crosstalk demonstrates that biological function depends on communication across organs and scales (Bartsch et al., 2015; Priest et al., 2019). Similarly, organ-on-a-chip and microphysiological systems research demonstrates that while individual tissues can be modeled with increasing fidelity, connecting these systems introduces new challenges, including scaling, timing, and the accurate representation of inter-system signaling (Ingber, 2022; Low and Tagle, 2021; Marx et al., 2016). These challenges are not unique to biological modeling. As AI-enabled tools are increasingly integrated into biotechnology workflows, similar difficulties arise in connecting models across levels of abstraction. In both cases, the central challenge shifts from improving individual components to understanding and designing the relationships between them.

A central limitation of siloed approaches is not simply that they isolate components, but that they obscure the relationships that govern system behavior. In biological systems, these relationships are often nonlinear, context-dependent, and mediated across multiple spatial and temporal scales. In AI-enabled biotechnology, these relationships are often implicit and only partially characterized. System behavior is shaped not only by component performance, but by how components are coupled across spatial, temporal, and organizational contexts. As a result, the challenge of understanding or engineering such systems lies not only in characterizing individual parts, but in modeling how those parts are coupled across scales.

Evidence from systems biology underscores this point. Network-based approaches have shown that perturbations in one pathway can propagate across seemingly unrelated systems, producing emergent effects that are not predictable from local behavior alone (Barabási et al., 2011). Similarly, efforts to integrate microphysiological systems reveal that once components are connected, system behavior becomes highly sensitive to coupling parameters such as flow rates, scaling relationships, and timing (Marx et al., 2016). These interactions are not secondary features; rather, they are primary determinants of system function.

A similar dynamic is emerging in AI-enabled biotechnology. Models trained for specific tasks, such as sequence generation, structural prediction, or functional evaluation, are increasingly combined into workflows that span multiple levels of abstraction. However, the relationships between these models are often implicit and only partially characterized, with system behavior shaped by interactions that are difficult to predict or evaluate in isolation. Recent work in multi-agent AI systems suggests that risks and failure modes can emerge from these interactions, even when individual components perform as intended (Rahwan et al., 2019; Kovač et al., 2025). As a result, system behavior may be driven as much by how components are coupled across physical, spatial, and temporal contexts as by the performance of the components.

## Compositional risk in AI-enabled workflows

A key implication of connected systems is that risk may emerge through composition. As an example, nucleic acid synthesis screening provides a widely adopted safeguard. Providers screen sequences against databases of known pathogens and toxins using sequence similarity methods (Diggans and Leproust, 2019). This approach has proven effective for detecting known threats.

However, advances in generative biotechnology introduce new dynamics. Machine learning models can generate novel sequences that diverge substantially from known references while preserving functional properties (Madani et al., 2023; Rives et al., 2021), expanding the accessible design space beyond what similarity-based screening was designed to evaluate. In a typical AI-enabled workflow, a generative model proposes candidate sequences that are iteratively refined and evaluated across multiple models. The resulting outputs may differ significantly from known sequences while retaining biologically relevant behavior, creating conditions under which downstream safeguards may not fully capture system-level risk.

When submitted for synthesis, these sequences may not trigger safeguards if they lack sufficient similarity to known agents, since current screening approaches rely primarily on sequence homology to known threats (Diggans and Leproust, 2019). However, recent work demonstrates that machine learning models can generate functional biological sequences that extend beyond known reference sequences while preserving structural and biochemical properties (Madani et al., 2023; Rives et al., 2021). As a result, sequences with potentially significant biological function may not be detectable using similarity-based screening alone (National Academies of Sciences, Engineering, and Medicine, 2018; Carter et al, 2023).

In this example, risk does not arise from a failure of any individual component, but from the interaction between generative design systems and screening frameworks. This illustrates a broader pattern: safeguards that are effective in isolation may not compose when systems are integrated, because risk can emerge at the interfaces between otherwise functional components. While empirical demonstrations of this specific failure mode are still emerging, the underlying technical conditions, namely the ability of generative models to explore functionally relevant but previously unobserved regions of sequence space, combined with screening approaches based on similarity to known references, are now well established in the literature (Madani et al., 2023; Rives et al., 2021; National Academies of Sciences, Engineering, and Medicine, 2018; Carter et al, 2023). As generative models expand biological possibility, the distinction between known and unknown threats becomes increasingly difficult to maintain (National Academies of Sciences, Engineering, and Medicine, 2018; Carter et al, 2023). A relational approach would therefore prioritize explicit modeling of interfaces as sites of risk, preservation of uncertainty and provenance across components, cross-system validation rather than component-level evaluation, and monitoring of emergent behavior across workflows. These interventions shift emphasis from optimizing individual components to governing system behavior under composition. In contrast to conventional systems approaches, relational biosecurity explicitly treats interfaces, information flows, and cross-system interactions as primary sites of risk, requiring design and governance interventions at these points rather than only at the level of individual components.

This pattern is not unique to biological systems. Similar concerns are emerging in AI safety, where increasingly capable systems are constructed by combining models, tools, and data sources into multi-step workflows. Studies of large language models augmented with external tools, retrieval systems, and other agents suggest that capabilities may emerge through composition in ways that are not predictable from the properties of individual components alone (Rahwan et al., 2019). At the same time, current safety approaches, such as model evaluation, red-teaming, and access controls, are typically applied at the level of individual systems rather than across system-level interactions within the full workflow.

These observations suggest a broader limitation: safeguards that are effective in isolation may not compose when systems are integrated. The challenges observed in AI-enabled biology reflect a more general problem in AI safety, the need to understand and govern the behavior of connected systems, rather than only their constituent parts.

## The role and limits of human oversight

Human oversight remains essential but is not sufficient. Research in AI governance demonstrates that the effectiveness of human-in-the-loop approaches depends on system design, information flow, and institutional context (Rahwan et al., 2019; Floridi et al., 2018). In complex systems, it is possible that no single actor will have full visibility.

Work on meaningful human control further shows that oversight is only effective when individuals have both contextual understanding and the authority to act (de Santoni Sio and van den Hoven, 2018). In practice, human decision-making within complex systems is shaped by local incentives, institutional constraints, and incomplete information. A central limitation of current human-in-the-loop approaches is that alignment between local decision-making and system-level objectives is often assumed rather than explicitly defined, represented, and enforced. This introduces a distinct challenge: values must not only be specified, but encoded in forms that can persist and propagate across systems and organizational boundaries. Drawing on concepts from interoceptive inference (Seth et al., 2013), this suggests that systems may require internal representations of their own state, uncertainty, and deviations from expected behavior, enabling more adaptive and context-sensitive responses. Without a shared framework for evaluating system-level outcomes, individual actors may optimize for proximal goals that do not align with broader objectives.

Addressing this limitation requires moving beyond procedural oversight toward the development of shared, system-level definitions of desirable and undesirable outcomes. This includes not only defining values, but also developing methods to represent, measure, and propagate them across components. Without such alignment mechanisms, human oversight risks reinforcing system dynamics rather than guiding them.

These challenges also have implications for AI governance. Current governance approaches often focus on regulating access to models, defining acceptable uses, or establishing oversight mechanisms at the level of individual systems. Emerging regulatory frameworks, such as the European Union’s AI Act, begin to address system-level impacts by considering risk in the context of deployment and use. However, the effectiveness of these approaches in capturing risks that emerge dynamically across interconnected workflows remains an open question. As capabilities become increasingly distributed across workflows, responsibility for outcomes may also become distributed, with no single human having full visibility or control. This creates gaps in accountability and complicates efforts to ensure alignment with shared values.

Addressing these gaps will require governance frameworks that extend beyond individual components to consider how systems are assembled, how information flows between them, and how values are represented and enforced across these interactions. In this context, relational approaches to biosecurity may offer a useful model for broader AI governance, emphasizing the design and oversight of system-level behavior.

This does not diminish the importance of human oversight; rather, it highlights that its effectiveness depends on the design of the surrounding system, including what information is visible, how uncertainty is represented, what values are employed, and where authority is situated. This highlights the need for system-level alignment with explicitly defined values, including safety, reliability, and broader societal objectives, as well as visibility into how these values are represented and propagated across system components.

## Designing for connection: toward relational biosecurity

If risk emerges from relationships, then safety must be designed at the relationship level. In biological systems, organisms maintain stability through distributed regulatory networks that sense and respond to perturbations, enabling robustness and equilibrium (Kitano, 2004; Friston, 2010). In this context, buffering refers to mechanisms that limit the propagation or amplification of errors, uncertainty, or unintended effects across system components. AI-enabled systems will also benefit from comparable system-level capabilities, including the ability to monitor interactions across components, preserve context, and model second-order effects. A relational biosecurity approach would treat interactions as first-class objects of design and governance. This includes monitoring interactions, preserving context, and aligning system behavior with shared values. Examples include tracking provenance across workflows, preserving uncertainty across interfaces, implementing cross-system validation, and detecting patterns of interaction rather than isolated outputs. This perspective is consistent with principles from control theory and cybernetic approaches to system management, which emphasize feedback, monitoring, and adaptive response in complex systems.

A relational approach to biosecurity emphasizes designing systems that explicitly account for interactions between components, including how information, uncertainty, and control signals propagate across workflows and their interfaces. This includes not only robustness to perturbations, but also the ability to detect, interpret, and respond to changes in system behavior as they arise. In this sense, relational design extends beyond traditional notions of robustness to include continuous system awareness and adaptive response. It also requires aligning system behavior with shared values, extending beyond component-level safeguards. In practice, this could include mechanisms such as tracking the provenance and transformation of information across workflows, preserving uncertainty and model confidence across interfaces, and implementing system-level monitoring that detects patterns of interaction rather than isolated outputs. These relationships are shaped by factors such as the rate of information exchange, the scale at which models and data are integrated, and the timing of interactions across workflows, all of which can significantly influence system behavior and the emergence of risk.

Developing relational biosecurity solutions does not require abandoning existing safeguards, but recontextualizing them within a system-level framework. Screening, access controls, and model evaluations remain essential, but must be complemented by mechanisms that account for how these elements interact. This may include designing workflows that preserve uncertainty across interfaces, implementing cross-system validation processes, and developing monitoring approaches that detect emergent patterns of behavior rather than isolated outputs.

Relational biosecurity is not a replacement for current approaches, but an extension that recontextualizes existing safeguards within connected systems.

## Research directions

Advancing a relational approach to biosecurity requires new lines of inquiry that explicitly address the behavior of connected systems. A priority is the development of methods to map and characterize AI-enabled biological workflows as integrated systems rather than discrete components. This includes identifying points of interaction among models, data sources, and experimental processes; characterizing how information, uncertainty, and error propagate across these connections; and analyzing the rates, scales, and timing of exchanges between components. Such mapping efforts would provide a foundation for identifying potential points of failure that are not visible at the level of individual tools.

A second area of research concerns the development of metrics and evaluation frameworks for system-level performance. Current evaluation practices focus largely on the accuracy or safety of individual models. However, as workflows become increasingly compositional, there is a need for metrics that capture properties such as robustness to perturbation, preservation of uncertainty across interfaces, and the emergence of unintended capabilities through multi-step processes. System-level evaluations could include robustness to perturbation, preservation of uncertainty across interfaces, detectability of emergent failure modes, and divergence between local and global objectives. Developing such metrics will require integrating insights from systems biology, control theory, and complex systems science, and adapting them to the context of AI-enabled biological design. Given the breadth of potential system-level properties, prioritization will be essential. Initial efforts may focus on high-impact areas such as robustness to perturbation and the detection of unintended capability emergence, where risks are both significant and tractable to measure.

A third priority is the study of compositional failure modes. While risks associated with individual technologies have been extensively studied, less is known about how failures emerge when systems are combined. This includes understanding how small biases or errors in one component may be amplified through downstream interactions, how task decomposition may obscure intent across workflows, and how distributed systems may enable capabilities that exceed those of any individual component. Experimental and simulation-based approaches could be used to systematically explore these dynamics, providing empirical grounding for system-level risk assessment.

A fourth area of focus is the integration of human and institutional factors into system design. As AI-enabled biological systems become more complex, human oversight will depend on how information is presented, how uncertainty is represented, and how decision-making authority is structured. Research is needed to develop interfaces, governance mechanisms, and organizational models that support meaningful human engagement at the system level. This includes defining and operationalizing shared values, and ensuring that these values are consistently represented across components and workflows.

These research directions should be interpreted across different categories of risk. In deliberate misuse scenarios, relational analysis can help identify how individually compliant tools may be combined into harmful workflows. In accidental harm scenarios, it can help trace how errors or uncertainty propagate through poorly characterized interfaces. In structural risk contexts, it can help examine how incentives and system design produce undesirable outcomes without a discrete point of failure.

Finally, there is a need to develop architectures for system-level sensing and feedback. Biological systems maintain stability through distributed mechanisms that monitor internal state and respond to perturbations. Analogous capabilities in AI-enabled biotechnology systems could include monitoring patterns of system-level interactions across workflows, detecting deviations from expected behavior, and enabling adaptive responses. In practice, this might involve combining workflow tracing, uncertainty propagation, and anomaly detection methods to identify when system behavior diverges from expected patterns. While such approaches remain technically challenging, they offer a pathway toward dynamic, system-aware forms of biosecurity that extend beyond static safeguards. Cybersecurity practices, including shared threat intelligence and coordinated response frameworks, may offer useful models for developing system-level sensing and feedback in AI-enabled biology, particularly where risks cross organizational boundaries.

Together, these directions suggest a shift from evaluating components in isolation to understanding and designing the behavior of connected systems, positioning biosecurity as a problem of managing interactions under uncertainty.

Addressing these challenges will require not only technical advances, but new forms of coordination across disciplines and sectors. The development of relational approaches to biosecurity depends on bringing together expertise from AI engineering, biological sciences, biosecurity, cybersecurity, ethics, human-centered design, and public policy, including stakeholders from academia, industry, and government.

One potential path forward is the establishment of collaborative chartering processes that define shared goals, principles, and research priorities for connected AI-enabled biological systems. Such efforts could draw on lessons from prior cross-disciplinary initiatives, while recognizing that the scale and complexity of current systems introduce new challenges. By creating spaces for sustained collaboration and shared problem definition, these processes could help align technical development with system-level understanding of risk and responsibility.

## Conclusion

AI-enabled biotechnology is transforming how biological systems are designed and governed. The central challenge is not only controlling individual technologies but governing how they interact. By treating interactions as explicit objects of design and aligning system behavior with shared values, relational biosecurity provides a framework for addressing compositional risk while supporting responsible innovation.

These challenges are not domain specific. As AI systems across domains become increasingly interconnected, the emergence of compositional risk and the limitations of component-level safeguards are likely to become more pronounced. Approaches that emphasize relational design, system-level sensing, and alignment across interactions may therefore have broader relevance for AI safety and governance.

By focusing on how systems are connected, rather than only on what individual components can do, it may be possible to develop frameworks that better account for the complexity of real-world deployments.

## Data Availability

The original contributions presented in the study are included in the article/supplementary material, further inquiries can be directed to the corresponding author.
